# ﻿A new species of *Notacanthella* Jacobus & McCafferty, 2008 (Ephemeroptera, Ephemerellidae) from Yunnan, China

**DOI:** 10.3897/zookeys.1103.82984

**Published:** 2022-05-26

**Authors:** Xian-Fu Li, Ye-Kang Sun, Zi-Ye Liu, Luke M. Jacobus, Wen Xiao

**Affiliations:** 1 Institute of Eastern-Himalaya Biodiversity Research, Dali University, Dali 671000, Yunnan, China; 2 Division of Science, Indiana University Purdue University Columbus, Columbus 47203, IN, USA; 3 Collaborative Innovation Center for Biodiversity and Conservation in the Tree Parallel Rivers Region of China, Dali University, Dali, Yunnan, China; 4 Research Center of Ecology and Governance for Er’hai Lake Streams, Dali, Yunnan, China

**Keywords:** Cangshan Mountain, Hengduan Mountains, Mayfly, Southwest China

## Abstract

*Notacanthellajinwu* Li & Jacobus, **sp. nov.** is described based on egg, nymph, and winged stages from Dali Bai Autonomous Prefecture, Yunnan Province, China. The nymph of the new species is closely related to *N.commodema* (Allen, 1971), whose nymphs share a similar tuberculation of head, pronotum, and mesonotum. However, the nymph of our new species can be distinguished based on the structures of male sternum IX and abdominal tergal tubercles. In addition, the new species is distributed in subtropical high-altitude areas. The description of the male imago of the new species is the first certain one for the genus *Notacanthella*. Data associated with our new species allow for expanded discussion and diagnosis of *Notacanthella* and closely related genera. An identification key for nymphs of these groups is provided.

## ﻿Introduction

Jacobus and McCafferty (2008) were the last to revise the genera of the mayfly family Ephemerellidae (Ephemeroptera). Their “*nigra* group” (Jacobus and McCafferty 2008: fig. 94) included five eastern Palearctic and Indomalayan genera: *Adoranexa* Jacobus & McCafferty, 2008, *Cincticostella* Allen, 1971, *Ephacerella* Paclt, 1994, *Notacanthella* Jacobus & McCafferty, 2008, and *Spinorea* Jacobus & McCafferty, 2008. Subsequent studies have emphasized the relationships of these groups but have had limited taxon sampling (Ogden et al. 2009; Zhang et al. 2021). Considerable contributions have been made in the last few years to our knowledge of the genera *Cincticostella* and *Notacanthella* (Martynov et al. 2019, 2021; Auychinda et al. 2020a, b; Li et al. 2020; Zhang et al. 2020, 2021; Zheng and Zhou 2021).

Based on the work reviewed above, *Cincticostella* now contains 21 species, *Notacanthella* and *Spinorea* each contain three species, and *Adoranexa* and *Ephacerella* are monospecific.

The genus *Notacanthella* has been reported low altitude areas of China, Thailand, and Vietnam, and it currently is comprised of the following species: *Notacanthellacommodema* (Allen, 1971), *N.perculta* (Allen, 1971), and *N.quadrata* (Kluge & Zhou in Kluge et al. 2004). Two other species attributed to this genus that were known only as male imagos were recently confirmed to be conspecific with a related species, *Cincticostellagosei* (Allen, 1975) (Zhang et al. 2021). At one time, *Notacanthella* species were divided into two subgenera. However, this classification was revised based on new observations of the lateral serration of the maxillary canines, which is prone to wear and is often difficult to examine. As a result, the subgenus Samiocca Jacobus & McCafferty, 2008 is considered to be a strict synonym of *Notacanthella* (Auychinda et al. 2020b). The imago stages of the three *Notacanthella* species remain unknown, and the egg is known only for *N.quadrata* (Auychinda et al. 2020b: fig. 7). Zhang et al. (2021) emphasized the need for research on this group and both Zhang et al. (2021) and Martynov et al. (2021) raised questions about the relationships of species within *Cincticostella* and among related genera.

During our recent survey of the mayfly fauna of Hengduan Mountain Area, southwest China, an undescribed species of *Notacanthella* was found only in high altitude areas. Here, we describe this new *Notacanthella* species based on imago, subimago, nymph, and egg stages. Our laboratory association of the male imago provides the basis for the first confident description of the male imago of *Notacanthella*.

## ﻿Materials and methods

*Notacanthella* nymphs were collected with a D-frame net from moderately fast-flowing areas of streams in Dali Bai Autonomous Prefecture, western Yunnan, China. Habitat photographs were taken using a Huawei Nova 8 mobile phone equipped with a Kase 40–75 mm macro lens. Some specimens were dissected under the stereomicroscope and were mounted on slides with Hoyer’s solution for examination under the digital microscope. Slide-mounted specimens were examined and photographed under a Keyence VHX-S550E digital microscope. For scanning electron microscopy (SEM), eggs were dried, coated with gold, and observed with a VEGA3 SBU SEM (Tescan, Brno, Czech Republic). Measurements were taken using ImageJ image processing software. Final plates were prepared with Adobe Photoshop CC 2018.

All materials examined of the new species are deposited in the Museum of Biology, Institute of Eastern-Himalaya Biodiversity Research, Dali University, Dali, Yunnan, China (**MBDU**).

The map of the sampling sites was made in QGIS Standalone Installer v. 3.10 and the 30-m Digital Elevation Model (DEM) data is provided by Geospatial Data Cloud site, Computer Network Information Center, Chinese Academy of Sciences (http://www.gscloud.cn).

We utilized a combination of morphological and ecological species concepts when formulating species hypotheses.

## ﻿Results

### ﻿ Notacanthella jinwu

Taxon classificationAnimaliaEphemeropteraEphemerellidae

Li & Jacobus sp. nov.

BF33CDC4-2123-5A7B-A83E-57B2A50EF291

http://zoobank.org/9C0000D1-7B7E-4367-BC67-0548820E97DD

Figs 1 , 2 , 3 , 4 , 5 , 6 , 7 , 8 , 9 , 10

#### Material examined.

***Holotype***: male, with final nymphal instar exuvia (in ethanol, deposited in MBDU), China, Yunnan Province, Dali City, Mt. Cangshan, Mocan Stream, 25°39'22.2"N, 100°11'10.1"E, 2020 m a.s.l., 23.X.2021, coll. Xian-Fu Li. ***Paratypes***: 10 nymphs, 6 imagos and 3 subimagos reared from nymphs with same data as holotype; 10 nymphs and 4 imagos reared from nymphs from same location as holotype, but 23.X.2021, coll. Xian-Fu Li; 20 nymphs and 5 imagos reared from nymphs from type locality, but 19.IX.2021, coll. Xian-Fu Li; 1 nymph, Dali City, Mount Cangshan, Qingbi Stream, 25°39'05.5"N, 100°9'08.4"E, 2316 m a.s.l., 14.V.2021, coll. Kun Yang; 3 nymphs, Qingbi Stream, 25°40'11.0"N, 100°11'02.7"E, 1974 m a.s.l., 3 nymphs, Qingbi Stream, 25°39'20.2"N, 100°9'44.1"E, 2098 m a.s.l., 16.VIII.2021, coll. Kun Yang; 3 nymphs, Qingbi Stream, 25°39'08.6"N, 100°9'27.3"E, 2221 m a.s.l., 21.VIII.2021, coll. Kun Yang; 2 nymphs, Yunnan, Bincuan City, Mount Jizushan, Shazhi River, 25°56'54.4"N, 100°21'40.0"E, 1947 m a.s.l., 21.VIII.2021, coll. Rong-Long Yang and Kun Yang. All the specimens are deposited in MBDU.

#### Diagnoses.

The new species is similar to *N.commodema* because both have nymphs with two pairs of flattened tubercles on the head, genae that are not produced into sharp projections, seven prominent tubercles on the pronotum, seven tubercles on the mesonotum, claws of all legs with five or six basal denticles, and posterolateral projections of abdominal segment IX that are not elongate. The new species can be distinguished from *N.commodema* by the shape and orientation of its longer and sharper abdominal tergal tubercles and by the structure of abdominal sternum IX in males, which is subquadrate with rounded posterolateral projections (see identification key, below). The ecological distribution of our new species is in subtropical high-altitude areas, in contrast to *N.commodema*, which is found in areas below 1000 m elevation. The imagos of other *Notacanthella* species are not known, so a diagnosis is not possible. Likewise, a meaningful diagnosis of the egg stage is not possible, either. See discussion for further information and remarks.

#### Descriptions.

**Figure 1.**
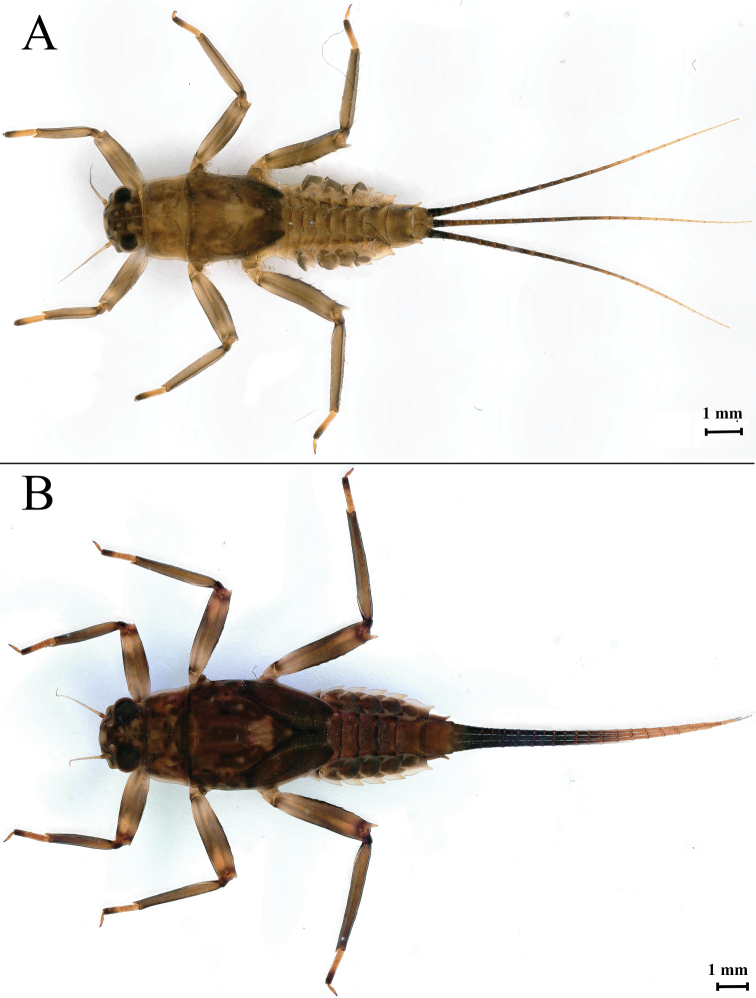
*Notacanthellajinwu* Li & Jacobus, sp. nov. **A** middle instar, dorsal habitus **B** last nymphal instar, dorsal habitus.

***Final nymphal instar*** (in ethanol). Body length 12.08–12.30 mm (excluding tails); head width 2.15–2.54 mm, cerci lengths 9.04–10.50 mm, median filament 9.57–10.60 mm. Body coloration brown with dark brown markings (Fig. 1A, B).

**Figure 2.**
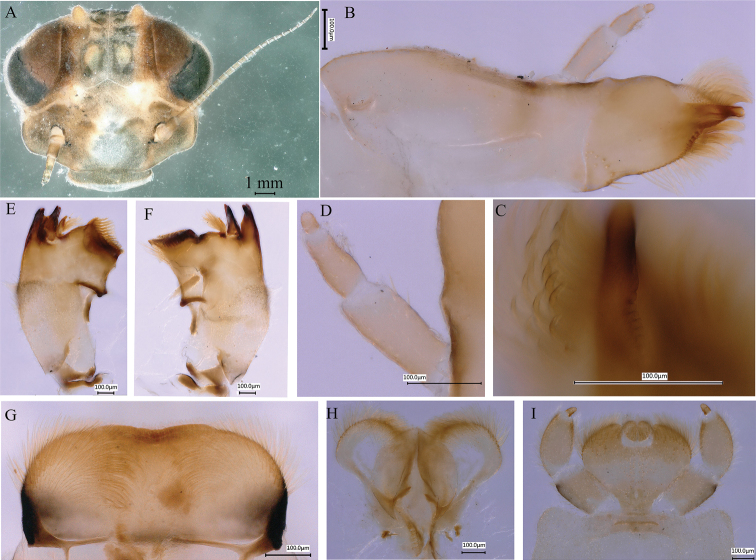
*Notacanthellajinwu* Li & Jacobus, sp. nov. **A** head, anterior view **B** maxilla **C** maxillary canines with lateral serration **D** maxillary palp **E** left mandible **F** right mandible **G** labrum **H** hypopharynx **I** labium.

***Head*.** Brown, with two pairs of tubercles; large occipital tubercles and small suboccipital tubercles (Fig. 2A). Maxillae with maxillary canine length greater than relative width (Fig. 2B), and with lateral serration (Fig. 2C); three-segmented maxillary palp covered with hair-like setae, segment length ratio from base to apex = 3.1: 2.4: 1 (Fig. 2D). Left mandible (Fig. 2E) and right mandible (Fig. 2F), with three outer incisors and two inner incisors, with tuft of short setae present in concavity close to molar area, and densely covered with irregularly ordered hair-like setae on dorsolateral surface. Labrum densely covered with setae, anterior margin somewhat concave medially (Fig. 2G). Hypopharynx: sublingua rounded with anterolateral hair-like setae, lingua oval with anterolateral, short setae (Fig. 2H). Labium densely covered with hair-like setae and with transverse stripes; glossae length greater than width; labial palp three-segmented, first and second segments subequal in length, third segment smaller (Fig. 2I).

**Figure 3.**
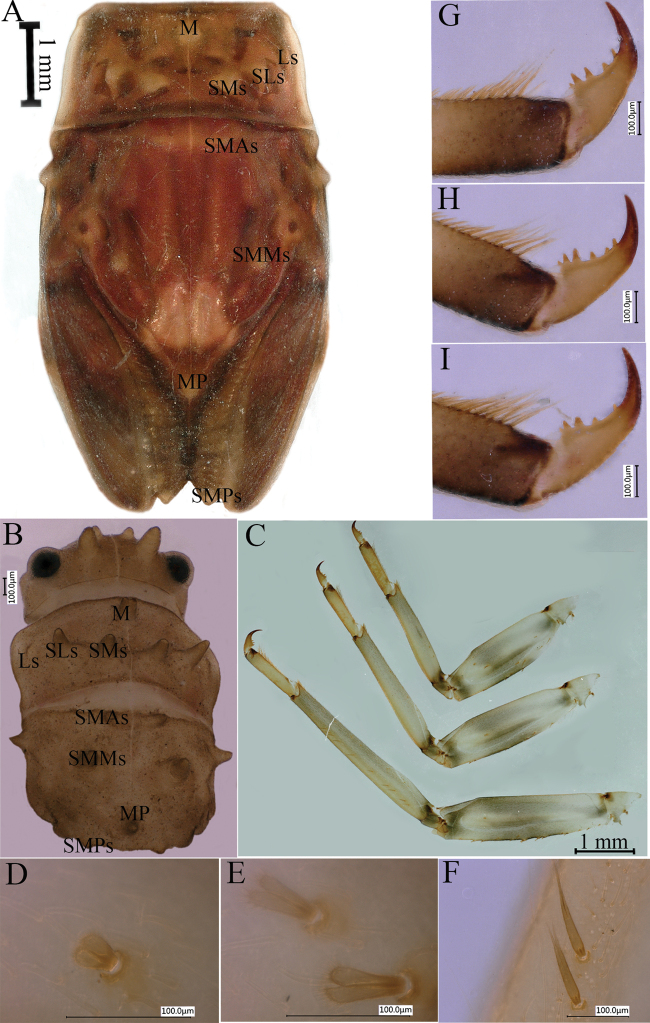
*Notacanthellajinwu* Li & Jacobus sp. nov. **A** thorax of last nymphal instar, dorsal view **B** thorax of early instar, dorsal view **C** legs, dorsal view, from top to bottom foreleg, midleg and hindleg **D** setae on femur **E** setae on femur **F** setae on tibia of hind leg **G** claw of foreleg **H** claw of midleg **I** claw of hindleg. (M=median tubercle; SMs = submedian tubercles; Ls = lateral tubercles; SLs = sublateral tubercles; SMAs = submedian anterior tubercles; SMMs = submedian tubercles at middle; MP = median posterior tubercle; SMPs = submedian posterior tubercles).

***Thorax*.** Pronotum without anterolateral projections; lateral margins convex; dorsal surface with seven tubercles: one medially, two submedially, two laterally, and two sublaterally; lateral tubercles prominent, but sublateral tubercles inconspicuous (Fig. 3A, B). Mesonotum with paired small and rounded anterolateral projection; lateral margins convex; dorsal surface with seven tubercles: two anteromedially, two medially, and three posteromedially (Fig. 3A, B). Foreleg: femur brown with dark brown bands medially and distally; dorsal margin with chalazae, short fine setae, and a few stout, pinnate, and clavate setae; ventral and outer margins densely covered with short, fine setae and few stout pinnate and clavate setae; dorsal and ventral aspects of tibia and tarsi brown with short, fine setae, few short, stout, pinnate, and clavate setae; apex of tibia and inner margin of tarsi with set of acute setae; ratio of femur: tibia: tarsus = 2.0: 1.9: 1 (Fig. 3C). Middle leg similar to foreleg, but ratio of femur: tibia: tarsus = 2.3: 2.5: 1 (Fig. 3C). Hind leg similar to foreleg and middle leg, but ratio of femur: tibia: tarsus = 2.5: 3.0: 1 and outer margin of tibia with row of long, stout, pinnate, and clavate setae (Fig. 3F). Long hair-like setae densely distributed at base of outer margin of each femur. Various stout setae of different lengths, some pointed and some rounded, present at apex of each tarsus (Fig. 3G–I). All claws with one row of five or six denticles (Fig. 3G–I).

**Figure 4.**
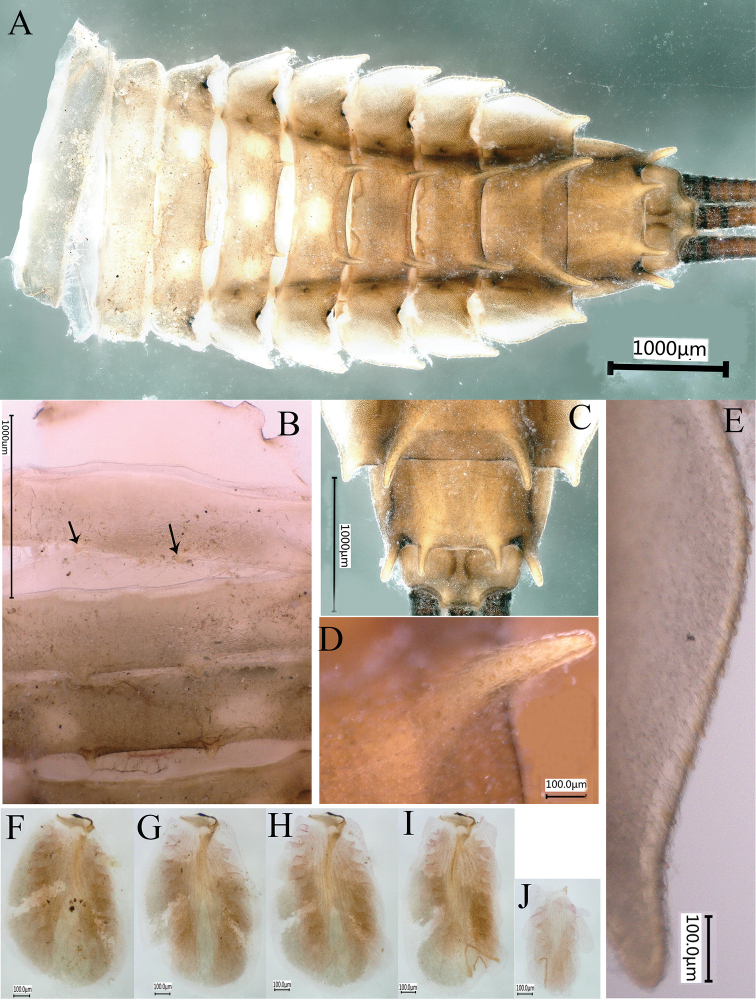
*Notacanthellajinwu* Li & Jacobus sp. nov. **A** abdomen, nymph, dorsal view **B** abdominal segments I–III of last nymphal instar **C** abdominal segments VII–X of last nymphal instar **D** tubercle of abdominal tergum VII of last nymphal instar **E** lateral margins of abdominal segment VII of last nymphal instar **F** gill III **G** gill IV **H** gill V **I** gill VI **J** gill VII.

**Figure 5.**
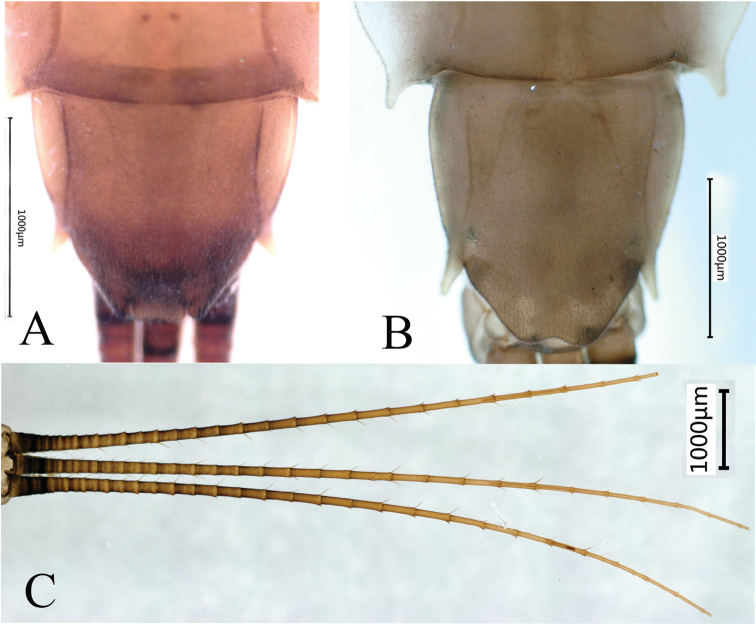
*Notacanthellajinwu* Li & Jacobus sp. nov. **A** structure of sternum IX of male nymph **B** structure of sternum IX of female nymph **C**nymphal caudal filaments.

***Abdomen*.** Abdominal terga brown, convex; terga III–VIII with prominent wing-like lateral projections (Fig. 4A); paired dorsal tubercles on segments I–X, tubercles short and tips parallel at base of segments I–IV, longer and tips progressively divergent on segments V–IX, long and divergent tubercles on segment IX, shorter and tips parallel on segment X; lateral projections of segment IX not extending beyond segment X (Fig. 4A, C). Lateral projections and apices of tubercles of each segment with stout, clavate setae (Fig. 4D, E). Posterior margin of sternum IX of male straight (Fig. 5A); posterior margin of sternum IX of female concave (Fig. 5B). Gills III–V with bifurcate and multifoliate ventral lamellae, gill VI ventral lamella integral and multifoliate, gill VII ventral lamella multifoliate; dorsal lamella of gill III rounded (Fig. 4F), dorsal lamellae of gills IV–VII paddle-shaped (Fig. 4G–J). Caudal filaments brown with whorls of small, almost rounded, scale-like setae and few long, unbranchedsetae at apex of each segment (Fig. 5C).

#### Character variability.

We examined specimens of different instars and some characters may vary between earlier and later instars, similar to its close relative, *N.commodema* (Allen 1971; Auychinda et al. 2020b).

**Figure 6.**
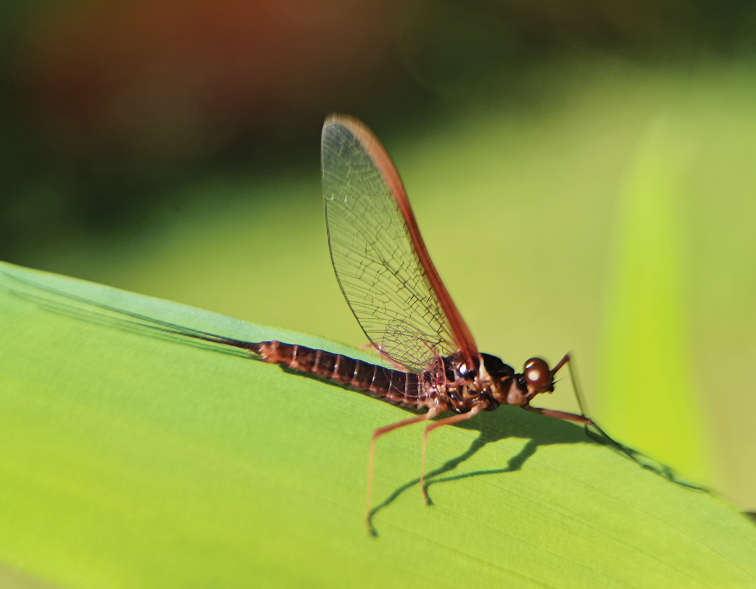
Male imago of *Notacanthellajinwu* Li & Jacobus sp. nov. (living).

**Figure 7.**
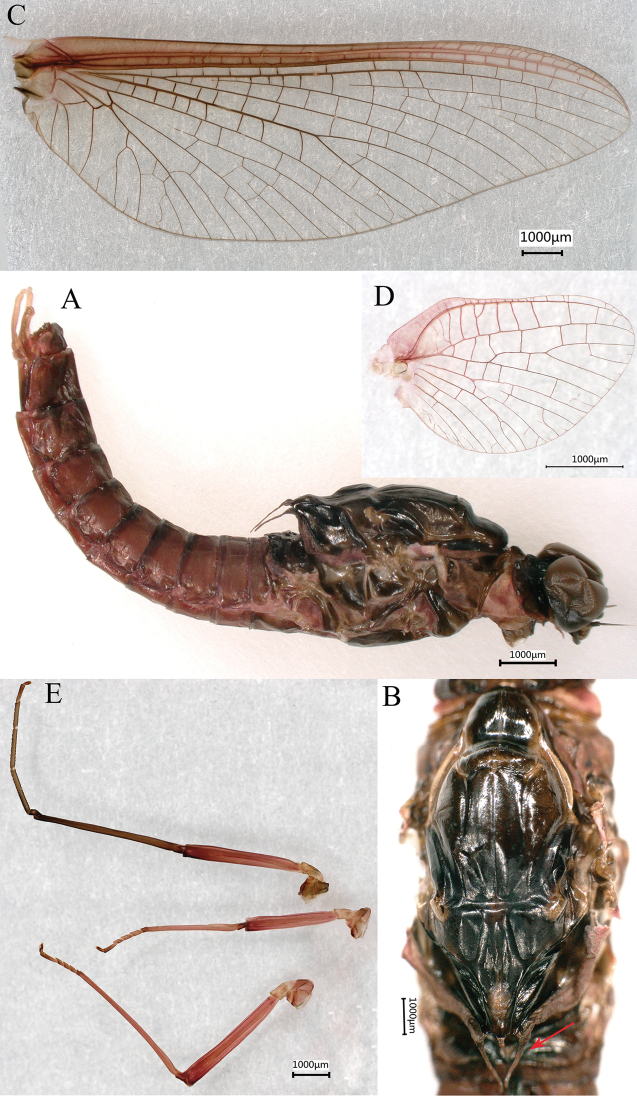
Male imago of *Notacanthellajinwu* Li & Jacobus sp. nov. **A** lateral view of body **B** dorsal view of thorax (lateral scutellar projection indicated by red arrow) **C** forewing **D** hindwing **E** legs, from top to bottom foreleg, midleg and hindleg.

***Male imago*** (in ethanol). Body length 11.73–13.17 mm (excluding tails), head width 2.13–2.36 mm, cerci lengths 11.31–13.98 mm, median filament length 11.07–14.52 mm, forewing length 14.12–17.31 mm, hindwing length 3.92–6.88 mm. Compound eyes contiguous, upper portion reddish brown and lower portion black. Body generally reddish brown to dark brown (Fig. 6). Prosternum dark brown, with slightly concave central longitudinal carina. Mesonotal scutellum with three projections at posterior margin, middle projection short (Fig. 7B). Forewings generally hyaline, veins reddish brown; cells of costal and subcostal fields tinted with reddish brown; cross veins in stigmatic area slightly oblique, and those between costal and subcostal separated into two rows cells; MA forked 1/4 distance from base to margin; MP forked 2/3 distance from base to margin (Fig. 7C). Hindwings hyaline, veins reddish brown; leading margin slightly concave; MA single, MP margin forked symmetrically (Fig. 7D). Fore legs reddish brown to dark brown, middle and hind legs reddish brown (Fig. 7E). All legs without distinct markings. Femur: tibia: tarsus of foreleg = 1: 1.3: 1.2, tarsal segments from basal to apical = 1: 3.5: 3.1: 2.0: 1.4; femur: tibia: tarsus of midleg = 1.8: 2.1: 1.0, tarsal segments from basal to apical = 1: 1.9: 1.8: 1.4: 2.3; femur: tibia: tarsus of hindleg = 2.8: 3.6: 1, tarsal segments from basal to apical = 1: 1.9: 1.8: 1.4: 2.3. Claws of all legs similar, one blunt and one hooked. Abdomen reddish brown to dark brown, terga I–VII each with one or three longitudinal median pale stripes; terga VIII–IX each with large and irregular pale stripes, posterolateral projections of terga VIII–IX each extended into sharp spine-like structures.

**Figure 8.**
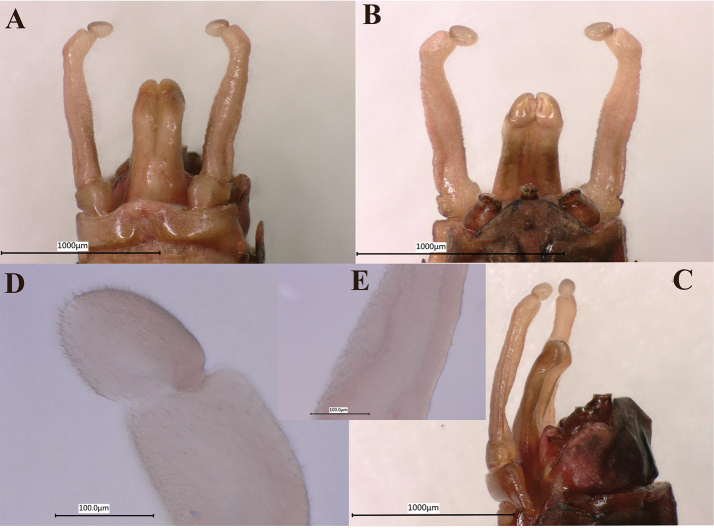
Male imago genitalia of *Notacanthellajinwu* Li & Jacobus sp. nov. **A** ventral view **B** dorsal view **C** lateral view **D** forceps segments 2 and 3 **E** bottom of forceps segment 2.

***Genitalia.*** Forceps covered with stout setae (Fig. 8D, E); segment 3 globular; segment 2 angled inward distally and with slight subapical constriction (Fig. 8A, B). Penes lobes compact, with linear groove on ventral face; lobes separated by slight cleft; anteromedial, dorsomedial and lateral stout setae absent; dorsolateral projection absent (Fig. 8A–C).

**Figure 9.**
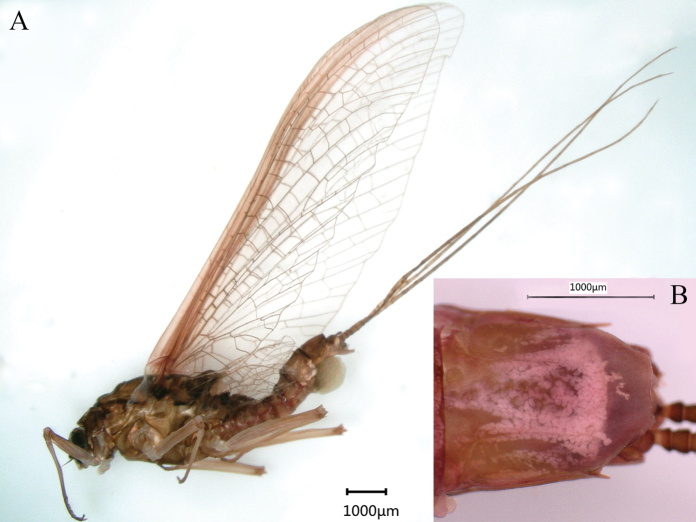
Female imago of *Notacanthellajinwu* Li & Jacobus sp. nov. **A** lateral view **B** terminal parts of abdomen, ventral view.

***Female imago*.** Colour pattern similar to male; body general reddish brown to dark brown (Fig. 9A). Body length 8.08–14.1 mm (excluding tails), head width 1.65–2.2 mm, cerci lengths 10.08–10.32 mm, median filament length 9.66–11.3 mm. Prosternum reddish brown, with slightly convex central longitudinal carina. Mesonotum dark brown; scutellum with three projections at posterior margin, middle projection short. Forewing 13.28–16.5 mm, hyaline, with veins reddish brown; cells C and SC tinted with reddish brown. Hindwing 3.45–4.8 mm, totally hyaline, with veins reddish brown. Each leg reddish brown to dark brown; length of femur: tibia: tarsus of foreleg = 2.0: 1.8: 1, tarsal segments from basal to apical = 1.4: 1.7: 1.7: 1: 2.4; femur: tibia: tarsus of midleg = 2.6: 2.7: 1.0, tarsal segments from basal to apical = 1: 1.1: 1.2: 1: 2.1; femur: tibia: tarsus of hindleg = 3.1: 3.7: 1.0, tarsal segments from basal to apical = 1.1: 1: 1.2: 1.2: 2.2. Abdomen reddish brown to dark brown; subgenital plate produced to 1/5 length of sternum VIII; posterior margin of subanal plate without obvious median cleft (Fig. 9B).

**Figure 10.**
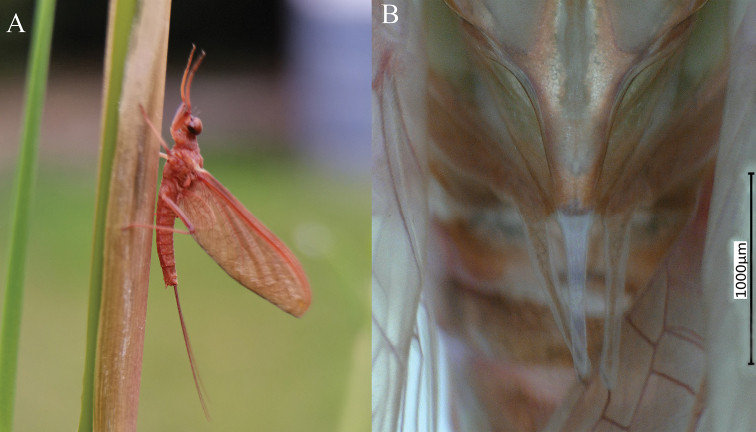
Male subimago of *Notacanthellajinwu* Li & Jacobus sp. nov. **A** living specimen **B** thorax, dorsal view.

***Male subimago*.** Body reddish brown (Fig. 10A); wings brown and subhyaline; scutellum with three long, pointed posterior prolongations (Fig. 10B); tarsus of foreleg shorter than femur, caudal filaments shorter than body length.

***Female subimago*.** Body red brown; wings brown and subhyaline; scutellum with three long, pointed posterior prolongations; tarsus of foreleg shorter than femur, caudal filaments shorter than body length. Posterior margin of subanal plate without obvious median cleft, similar to female imago. Otherwise, similar to male subimago except for usual sexual differences.

**Figure 11.**
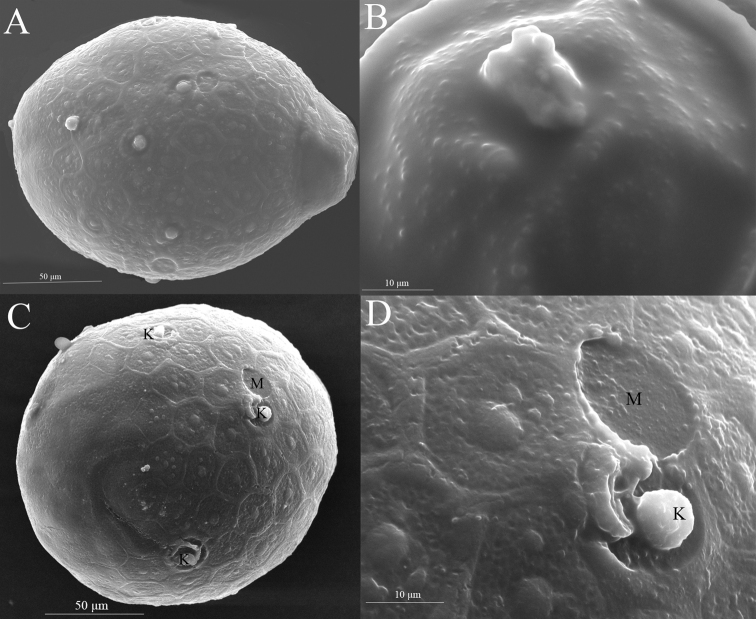
Egg of *Notacanthellajinwu* Li & Jacobus, sp. nov. **A** lateral view **B** polar cap **C** bottom view of the opposite pole **D** knob of attachment structure (K) and micropyle (M).

***Egg*** (dissected from female imago). Length 171–218 μm, width 134–158 μm. Ovoid with one small polar cap (Fig. 11A, B); chorion with reticulations, strands ridged; mesh with multiple central tubercles (Fig. 11A, C, D); several lateral attachment structures in subpolar areas (Fig. 11A, C); knob of attachment structure and micropyle (Fig. 11D) distributed near equator (Fig. 11A, C), micropyle round and micropylar rim absent.

#### Etymology.

The name, *jinwu* (feminine), comes from Jin Wu, a Chinese mythical creature. In China, ancient people took “Jin Wu” as the alias of the sun. The reddish brown subimago is similar to the color of a rising sun. Given that the emergence of *N.jinwu* sp. nov. happened at sunrise, we can imagine *N.jinwu* as the body double of the sun. The common name of this species is the Jinwu spiny crawler mayfly.

#### Distribution.

China (Yunnan).

#### Ecology.

**Figure 12.**
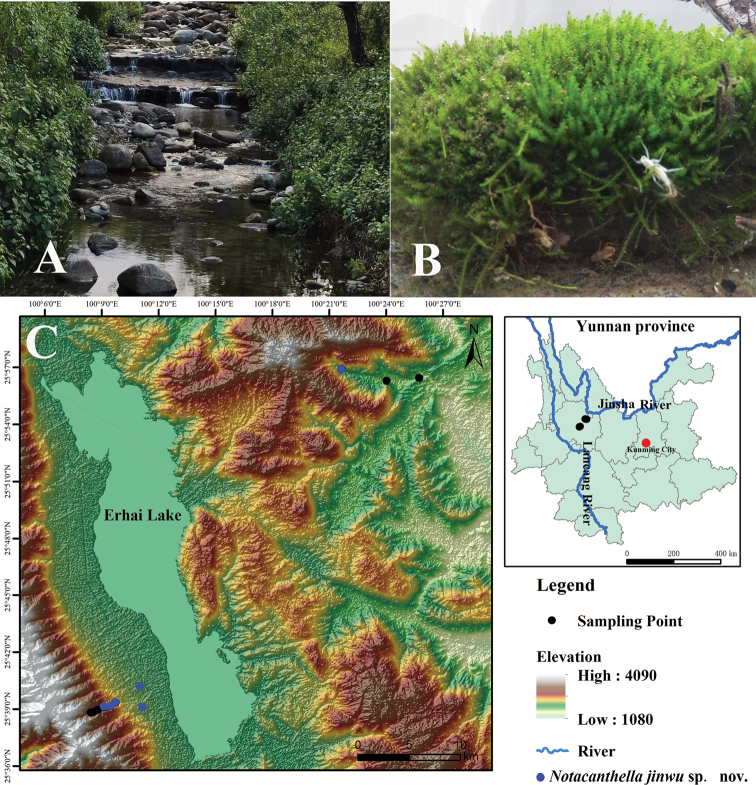
Habitat and distribution map of *Notacanthellajinwu* Li & Jacobus, sp. nov. **A, B** habitats in Dali City (note recently molted nymph in **B**) **C** distribution map of *N.jinwu* sp. nov. in Dali Bai Autonomous Prefecture.

The stream in Dali City and Binchuan County where the nymphs of *N.jinwu* were collected is 1.2–5.0 m wide, with a natural water body depth 5–35 cm. It contains stones of various sizes, aquatic plants, and litter (Fig. 12A). During collecting, the nymphs were found hiding under stones or climbing on aquatic plants, moving slowly and swimming weakly. The nymphs are only distributed between 1947 and 2316 m above sea level (Fig. 12C). In indoor conditions, nymphs generally hid under rocks (Fig. 12B), but they were more active when eating aquatic plants and litter. The last instar nymphs molted at sunrise and flew after a short rest. The subimago stage persisted for 2 days and molted during the daytime. The observed timespan of the imago stage was about 3 days. According to our monthly field survey, the nymphs of *N.jinwu* Li & Jacobus, sp. nov. are found from May to November.

## ﻿Discussion

Morphological plasticity within species is well documented for insects (e.g., Moczek 2010) and has been widely documented and assumed for mayflies. In our study, however, the morphology was remarkably unchanged between the early emergence individuals and the later ones. However, we did observe the morphological differences between instars, such as relative development of body armature, that have been documented elsewhere for this genus (e.g., Allen 1971; Auychinda et al. 2020b).

The different ecological niches of aquatic organisms inﬂuence their altitudinal patterns of distribution, based on differences in adaptability to the environment (Liu et al. 2021). In addition to morphological differences between our new species and *N.commodema*, there are also important differences in the ecological niches of the two species. *Notacanthellacommodema* is distributed in areas below 1000 m altitude in the tropics, but *N.jinwu* Li & Jacobus, sp. nov. is found in areas at altitudes around 2000 m in the subtropics.

A holistic approach is needed to address the systematics of *Notacanthella*, and so the related genera *Adoranexa*, *Cincticostella*, *Ephacerella*, and *Spinorea* are included in the discussion that follows, insofar as the state of knowledge allows.

The eggs of our new species (Fig. 11) have strands that are relatively smooth, in contrast to the strands of *N.quadrata* (Auychinda et al. 2020b: fig. 7) which are covered with excrescences and small papillae. The eggs of *N.commodema* and *N.perculta* are not known. The eggs of both *N.quadrata* and our new species differ from the egg of *Cincticostellagosei* (Zhang et al. 2021: fig. 6), which has a chorionic surface that lacks distinct strands; it is apparently roughened, with a variety of excrescences and a wrinkled appearance. *Spinoreamontana* (Kang & Yang, 1995) (Kang and Yang 1995: figs 9, 10) and several other *Cincticostella* species (e.g., Kang and Yang 1995: figs 14–17; Jacobus and McCafferty 2008: fig. 3) have eggs generally similar to our new species, but the eggs of *Spinoreaglebosa* (Kang & Yang, 1995) are different (Kang and Yang 1995: fig. 8). The eggs of *Spinoreagilliesi* (Allen & Edmunds, 1963) are not known. In *Ephacerellalongicaudata* (Ueno, 1928) (Ishiwata and Nishino 2020: figs 19, 20) the eggs have smooth strands and a rough, pitted mesh, extremely similar to many *Cincticostella* species. The eggs of *Adoranexasoldani* (Allen, 1986) are unknown. Some other ephemerellid genera that are not part of this group of genera also have similar eggs (e.g., Jacobus and McCafferty 2008: fig. 1; p. 245: key couplet 8), and thus this morphology may represent either a pleisiotypic or convergent condition.

The male genitalia of our new species (Figs 7A, 8) have a distal constriction on forceps segment 2. The forceps are constricted more distally than *Ephacerella* and some *Cinctictostella* species (Jacobus and McCafferty 2008; Zhang et al. 2021), but other *Cincticostella* species have a similar position of this constriction (e.g., Jacobus and McCafferty 2008: fig. 86). Worth noting, too, is that yet other *Cincticostella* species (Zheng and Zhou 2021: figs 6, 7) have forceps extremely similar to some *Ephemerella* Walsh, 1862 species (e.g., Jacobus and McCafferty 2008: figs 74, 75). Unfortunately, the other *Notacanthella* species and most *Cincticostella* species are not known in the male imago stage, nor are the genera *Adoranexa* and *Spinorea*. Thus, meaningful and informative comparisons are not possible, and few conclusions can be made at this time. Anecdotally, the male genitalia and coloration of our new species are very similar to how LMJ remembers the genitalia and appearance of *Notacanthella* sp. A of Jacobus and McCafferty (2008) from Thailand (Phitsanulok Province, Phu Hin Rongkla National Park; altitude 1280 m).

Commonly, the structure of nymphal sternum IX reflects the morphology of mayfly male genitalia developing underneath. So, we speculate that there will be male genitalia differences between the new species and *N.commodema.* However, since the imago of *N.commodema* remains unknown, this hypothesis remains untested. We note that Auychinda et al. (2020b) reported differences in sternum IX of female nymphs identifiable as *N.commodema*, and they considered the possibility of a cryptic species complex. More work, using different kinds of data, clearly is needed to investigate species diversity of *Notacanthella*.

Despite our fragmentary knowledge of the egg and male imago stages of this group of ephemerellid genera, all species are known in the nymphal stage. An updated key that would include all *Cincticostella* species is beyond the scope of this study. Eight *Cincticostella* species have been described since the last key was provided by Xie et al. (2009), and we are aware of several additional undescribed new species from western China alone. We do provide a key below, though, to all the species of *Adoranexa*, *Ephacerella*, *Notacanthella*, and *Spinorea* in order to facilitate recognition and further detailed studies of these species that might be easily confused with one another. Such detailed studies will help to resolve the poorly supported systematics of this group (Jacobus and McCafferty 2008; Ogden et al. 2009) and lead to evolutionary hypotheses important for understanding aquatic life in the Indomalayan region.

### ﻿Key to final nymphal instars of *Cincticostella*-complex genera and of species of *Adoranexa*, *Ephacerella*, *Notacanthella*, and *Spinorea*

**Table:** 

1	Pronotum with prominent anterolateral projections…*Cincticostella* and *Notacanthella* (in part)	**2**
–	Pronotum with anterolateral projections very subtle or absent	**3**
2	Maxillary canines reduced to short, denticulate blade	***Cincticostella***
–	Maxillary canines long and acute at apices	**8 (*Notacanthella)***
3	Lateral margins of abdominal posterolateral projections bare or with only a few, inconspicious setae (e.g., Fig. 4E); maxillary canines fused and distinctly spoonlike, with no notch at apex	**8 (*Notacanthella)***
–	Lateral margins of abdominal posterolateral projections with distinct setae; maxillary canines fused and either spoonlike with a single apical notch, or reduced to a wide blade	**4**
4	Lateral margins of mesal plate with paired spines or ridges	**5**
–	Lateral margins of mesal plate unadorned	***Ephacerellalongicaudata***
5	Maxillary canine blade length much less than width	***Adoranexasoldani***
–	Maxillary canine blade length subequal to width	**6 (*Spinorea)***
6	Maxillary palp long, tip nearly reaching apex of maxilla (Kang and Yang 1995: fig. 1E); abdominal tergal tubercles relatively short, not much longer than posterolateral projections of same abdominal segment (Kang and Yang 1995: fig. 1D)	***Spinoreaglebosa***
–	Maxillary palp relatively short, extending only to middle of galea-lacinia (Allen and Edmunds 1963: fig. 32; Kang and Yang 1995: fig. 2E); most abdominal tergal tubercles distinctly longer than posterolateral projections of same abdominal segment (Allen and Edmunds 1963: fig. 36; Kang and Yang 1995: fig. 2D)	**7**
7	Abdominal terga tubercles distinctly divergent (Kang and Yang 1995: fig. 2D); tarsal claw with 5–7 denticles (proximal denticle often tiny and easily overlooked)	***Spinoreamontana***
–	Abdominal tergal tubercles subparalellel (Allen and Edmunds 1963: fig. 36); tarsal claw with 2–4 denticles (proximal denticle often tiny and easily overlooked)	***Spinoreagilliesi***
8	Head with strong and acute genal projections; pronotum with distinct anterior projections	***Notacanthellaquadrata***
–	Head without strong genal projections; pronotum with anterior projections very subtle or absent	**9**
9	Posterolateral projections on abdominal segment IX extend well beyond posterior margin of segment X (Allen 1971: fig. 28)	***Notacanthellaperculta***
–	Posterolateral projections on abdominal segment IX do not extend beyond posterior margin of segment X (Figs 1A–B, 4A; Allen 1971: fig. 27)	**10**
10	Paired tubercles on terga VIII and IX short and blunt (Auychinda et al. 2020b: Fig. 4F); medial projection of male sternum IX short and rounded, with adjacent projections sharp at tips (see Auychinda et al. 2020b: fig. 5D)	***Notacanthellacommodema***
–	Paired tubercles on terga VIII and IX long and sharp (Fig. 4A, C); medial projection of male sternum IX longer and subquadrate, with adjacent projections rounded at tips (Fig. 5A)	***Notacanthellajinwu* sp. nov.**

## Supplementary Material

XML Treatment for Notacanthella jinwu
